# Predictive reducing sugar release from lignocellulosic biomass using sequential acid pretreatment and enzymatic hydrolysis by harnessing a machine learning approach

**DOI:** 10.1016/j.csbj.2025.09.027

**Published:** 2025-09-23

**Authors:** Suphitchayanee Namboonlue, Kittisak Ngowsakul, Kittiya Nakarat, Chatchol Kongsinkaew, Nakarin Subjalearndee, Pakorn Uttayopas, Theppanya Charoenrat, Tunyaboon Laemthong

**Affiliations:** a Department of Chemical Engineering, Faculty of Engineering, Thammasat School of Engineering, Thammasat University, Pathum Thani 12120, Thailand; b Department of Biotechnology, Faculty of Science and Technology, Thammasat University, Pathum Thani 12120, Thailand; c Advanced Composite and Nanotextiles Research Team, National Nanotechnology Center, National Science and Technology Development Agency, Phahonyothin Road, Khlong Nueng, Khlong Luang, Pathum Thani 12120, Thailand; d Department of Mechanical Engineering, Faculty of Engineering, Thammasat School of Engineering, Thammasat University, Pathum Thani 12120, Thailand

**Keywords:** Predictive system, Reducing sugars, Acid pretreatment, Enzymatic hydrolysis, Lignocellulose, Machine Learning

## Abstract

The development of sustainable production of bio-based chemicals is targeted towards the use of lignocellulose. Overcoming its recalcitrance is a crucial step for biomass valorization. Here, we demonstrate a predictive system for reducing sugar yield from acid pretreatment and enzymatic hydrolysis processes of two types of biomass, rice straw and sugarcane leaves, which have different lignocellulosic compositions. A machine learning model based on the Decision Tree algorithm was employed to predict the amount of reducing sugars generated during enzymatic hydrolysis. The model demonstrated satisfactory accuracy, with an R² of 0.8910 for the training set and 0.8121 for the testing set, along with low error values (RMSE 0.1042 and MAE 0.0705). Scanning electron microscope (SEM) revealed that the biomass structure undergoes significant changes after enzymatic hydrolysis, as proven by the formation of surface pores. This morphological alteration reflects the enzymatic degradation of cellulose, resulting from the disruption of fiber bonds. The application of machine learning in this research shows great potential for enhancing biomass conversion efficiency, contributing to biomass valorization efforts.

## Graphical Abstract

**Figure:**
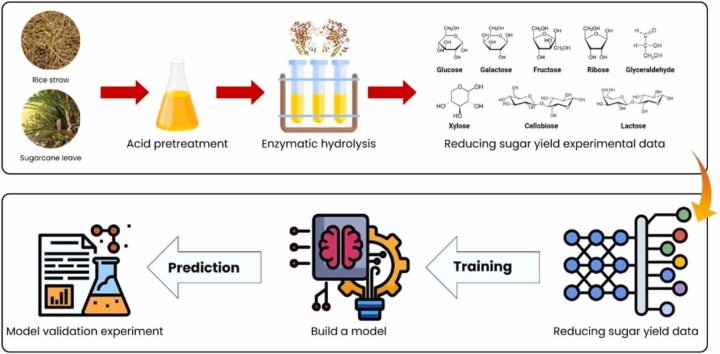

## 1 Introduction

Lignocellulosic materials are the most abundant and low-cost biomass available worldwide [1]. Especially in agricultural countries like Thailand, there is a diverse range of lignocellulosic raw materials. The main components of lignocellulosic biomass are cellulose, hemicellulose, and lignin [2]. The recalcitrance of this biomass makes it difficult to convert into chemicals. Therefore, saccharification technologies are crucial in liberating fermentable sugars. An efficient pretreatment method is necessary to ensure that sequential enzymatic hydrolysis maximizes sugar productivity while minimizing sugar loss [3]. In Thailand, around 25–27 million tons of rice straw and 15.47 million tons of sugarcane leaves are generated annually as residues [4], [5]. This study aims to valorize these lignocellulosic wastes in line with government initiatives promoting the use of agricultural by-products to add value, reduce waste, and mitigate environmental pollution [6]. In this study, rice straw and sugarcane leaves are selected as the primary lignocellulosic feedstocks, which have compositions as shown in Table 1.

**Table 1:** Lignocellulosic composition of rice straw and sugarcane leaves in Thailand [7], [8].

	**Cellulose**	**Hemicellulose**	**Lignin**
**Rice straw**	39.58	27.36	14.12
**Sugarcane leaves**	29.16	21.13	24.21

Several studies have utilized wheat straw (WS) as a lignocellulosic feedstock for reducing sugar production. However, its enzymatic hydrolysis rate of WS was relatively low due to the structural interference of lignin and hemicellulose. To enhance enzymatic accessibility, pretreatments with sodium hydroxide (NaOH) and sulfuric acid (H_2_SO_4_) were investigated. Among these, H_2_SO_4_ was particularly effective in removing hemicellulose, facilitating improved hydrolysis efficiency [9]. For example, at 2 % (w/v) H_2_SO_4,_ saccharification of rye straw achieved up to 69 % cellulose conversion in sodium citrate buffer [10]. Structural analysis indicated that both amorphous and partially crystalline cellulose regions were hydrolyzed when the combination of cellulase from *Trichoderma reesei* and cellobiase from *Aspergillus niger* was employed [9].

Many studies have optimized acid concentration and reaction conditions for higher yields. For example, pretreating rice straw with 1 % (w/w) H_2_SO_4_ at 160 °C to 180 °C for 1–5 min, followed by enzymatic hydrolysis, resulted in a sugar yield of 83 % [11]. Subsequent enzymatic hydrolysis using commercial enzyme, such as Celluclast 1.5 L (60 FPU/mL) and Novozyme 188 (285 IU/mL β-glucosidase activity), further improved sugar release due to increased biomass porosity. Pretreatment with organic acids has also shown promise. Lactic acid (LA), citric acid (CA), and acetic acid (AA) have been explored for sugarcane bagasse hydrolysis. Among these, monocarboxylic acids (LA and AA) were more effective than polycarboxylic CA. Specifically, AA pretreatment selectively removed lignin and disrupted cellulose structures, achieving 97.61 % glucan and 63.95 % xylan digestibility during subsequent enzymatic hydrolysis [12]. Additionally, hybrid approaches that integrate acid pretreatment with enzymatic hydrolysis have demonstrated significant potential for enhancing sugar yields. For example, pretreatment with acids, such as H_2_SO_4_ or organic acids (LA, AA), can remove hemicellulose and disrupt lignin–cellulose interactions, thereby increasing enzyme accessibility [11], [12]. Similarly, other chemical–biological hybrid methods, such as treatment with white liquor, a solution of sodium sulfide (Na_2_S) and sodium hydroxide (NaOH) at concentrations of 15–20 %, heated to 145–170 °C under 6–7 bar pressure for 2–3 h. The treatment effectively separates cellulose fibers, while the residual liquid, known as black liquor, contains solubilized lignin and hemicellulose components [13]. These findings demonstrate the synergistic effect of combining chemical pre-treatment with enzymatic hydrolysis to improve biomass conversion.

Acid pretreatment generally enables higher sugar extraction compared to alkaline methods. However, the need for high temperatures elevates energy requirements. To address this, it is of interest to pre-treat rice straw and sugarcane leaves with acid in combination with enzymatic hydrolysis, thereby modifying conventional acid processes to operate at lower temperatures. Identifying optimal conditions for maximum sugar yield remains a critical research goal. Integrating machine learning techniques can enhance this optimization by enabling predictive modeling of enzymatic efficiency and reducing experimental costs. Machine learning (ML) also facilitates real-time process control and efficient data interpretation, contributing to more sustainable bioconversion strategies [14].

Many studies have demonstrated the effectiveness of ML in predicting sugar yields from lignocellulosic biomass. For example, ANN has been successfully applied to predict sugar yields from alkali-pretreated rice straw during enzymatic hydrolysis, considering factors such as biomass loading and particle size, and identifying optimal hydrolysis conditions, including enzyme doses and reaction time [15]. Similarly, machine learning approaches have been used to predict xylose yields from hemicellulose hydrolysis in hardwoods, with ANN outperforming ridge regression, support vector regression, and traditional kinetic models, achieving a mean absolute error of 6.18 % [16]. Moreover, ANN has been shown to accurately model enzymatic hydrolysis kinetics of cellulose in heterogeneous systems, providing better predictions than response surface methodology and demonstrating its ability to mimic complex enzymatic reactions [17]. Beyond biomass hydrolysis, machine learning has also been increasingly applied in the design of green solvents such as ionic liquids (ILs) and deep eutectic solvents (DESs). These applications enable rapid prediction of solvent properties and pretreatment efficiency, reducing reliance on labor-intensive experimental screening [18]. By linking solvent design with hydrolysis performance, ML provides a useful framework for optimizing pretreatment strategies and maximizing sugar yields from lignocellulosic biomass.

ML can also help identify structure-property and composition-yield relationships from literature data, thereby supporting improved prediction and process optimization [19], [20]. In addition, ANN has been applied in specific optimization tasks, such as enhancing xylooligosaccharide production or ethanol yield under multi-objective constraints [21], [22], [23].

In this work, we aim to obtain predicted reducing sugar production from agricultural biomass using ML. To address the challenges posed by different lignocellulosic structures, we employed acid pretreatment followed by enzymatic hydrolysis. The hydrolysis step was performed using varying ratios of cellulase and xylanase, as these enzymes act synergistically. Cellulase breaks down cellulose into glucose, while xylanase degrades hemicellulose into xylose. Since rice straw and sugarcane leaves contain significant amounts of both cellulose and hemicellulose, the combined use of these enzymes enhances sugar release efficiency compared to using a single enzyme. The models were developed to predict sugar yields during the enzymatic hydrolysis of rice straw and sugarcane leaves.

## 2 Materials and methods

### 2.1 Raw materials and chemical reagents

Rice straw was collected from a local farm in Thailand. Sugarcane leaves were obtained as a gift from BIOTEC (NSTDA, Thailand Rice straw was collected from a local farm in Pathum Thani, Thailand, while sugarcane leaves were kindly provided by BIOTEC (NSTDA, Thailand). In addition, cellulase (13,000 U/mL) and xylanase (100,000 U/mL) were purchased from Reach Biotechnology, Pathum Thani, Thailand. All chemicals (AR grade) were purchased from KEMAUS Chemicals, Australia. The biomass was air-dried at 50 °C overnight and then sieved using a Retsch sieve to achieve a particle size to 30–45 mesh. The resulting material was dried at 50 °C to remove moisture. The dried materials were stored at room temperature until further use.

### 2.2 Acid pretreatment procedure

Sulfuric acid (98.08 g/mol, KEMAUS) solutions with concentrations of 0, 3, 6, and 9 %v/v were prepared and used to treat the samples, which were then heated in a water bath (Jubalo, Germany) at 60 °C with shaking at 100 rpm for 8, 16, and 24 h. After the designated time, the treated mixture was centrifuged using a centrifuge machine (MPW-380R, Poland) at 25 °C and 2000 rpm for 10 min to separate the biomass. The liquid fraction was collected and stored at 4 °C. The biomass was dried overnight at 50 °C and used for subsequent enzymatic hydrolysis.

### 2.3 DNS assay (3,5-dinitrosalicylic acid)

The procedure was modified from Bailey et al. [24]. Briefly, to prepare the 3,5-dinitrosalicylic acid (DNS) solution, 10 g of DNS was dissolved in 250 mL of distilled water. The solution was gradually added to 200 mL of 8 % NaOH while stirring the mixture on a hot plate until fully dissolved. Then, 300 g of potassium tartrate (C_4_H_4_K_2_O_6_) was added slowly while stirring continuously. The final volume was adjusted to 1000 mL by adding distilled water.

### 2.4 Enzymatic hydrolysis

The acid-pretreated biomass was used with a biomass/citrate buffer/enzyme ratio of 0.1/9.75/0.1 (g/mL/mL). Cellulase and xylanase were applied in varying combinations (100/0, 50/50, and 0/100), along with a control without enzymes (0 U/g solids). The total enzyme loadings of 1300 U/g solids for the combinations were used. All enzymes were supplied by iKnowenzyme (Reach Biotechnology, Pathum Thani, Thailand), and the citrate buffer was adjusted to pH 5.5. The mixture was incubated in a JULABO water bath at 50 °C while shaking at 150 rpm for 4, 16, 24, 32, and 48 h. After incubation, the mixture was centrifuged using a Centrifuge (MPW-38OR, MPW Med. Instrument, Poland) at 25 °C and 2000 rpm for 10 min to separate the biomass. The resulting liquid was used to measure the sugar content using the 3,5-Dinitrosalicylic acid (DNS) assay, as described previously.

### 2.5 Simple released sugar analysis

The reducing sugar content of the hydrolysate was determined using the 3,5-dinitrosalicylic acid (DNS) method, adapted from standard protocols. The liquid sample was first diluted 1–2 times with citrate buffer or further as needed to ensure the absorbance fell within the linear range of detection. From this diluted sample, 0.20 mL was transferred into a test tube, followed by the addition of 0.05 mL of NaOH (to enhance sugar reactivity) and 0.25 mL of DNS reagent. After heating, tubes were immediately cooled in cold water for 5 min to stabilize color development. Then, 5 mL of distilled water was added to each tube and mixed thoroughly. Absorbance was measured at 540 nm using a UV–VIS spectrophotometer (Model SP880, Metertech, Taiwan). The absorbance values of the unknown samples were compared to the standard glucose curve to determine the concentration of reducing sugars.

### 2.6 Sugar concentration prediction

To explore the potential of ML in predicting sugar concentration during enzymatic hydrolysis, experimental data obtained from Section 2.4 were used to develop preliminary predictive models. This dataset contains 84 samples, including enzyme percentages and corresponding sugar concentrations, obtained at hydrolysis durations of 4, 24, and 48 h. These 84 samples were generated by performing at least three replicates for each condition, under four cellulase/xylanase enzyme ratios (100/0, 50/50, 0/100, and 0/0). Seven attributes related to lignocellulosic composition and enzymatic hydrolysis parameters were selected as input features: percentages of cellulose, hemicellulose, and lignin (%); volumes of cellulase, xylanase, and citrate buffer (mL); and hydrolysis duration (hours). These parameters were selected because they represent the experimental variables that were systematically varied in this study. Each of these variables can potentially influence the output, and their inclusion allows the machine learning models to capture the effects of all relevant factors on reducing sugar concentration. The reducing sugar concentration, expressed in grams per gram of dry biomass (g/g dry biomass), was selected as the output feature. These input and output features were used to construct the dataset for this study.

For the predictive models, six common regression models were selected to capture a range of linear and nonlinear relationships between the experimental features and reducing sugar concentration: Linear Regression, Polynomial Regression, Decision Tree, Random Forest, Gradient Boosting, and Artificial Neural Networks. These models were chosen to compare simple interpretable models, ensemble methods, and neural networks, allowing evaluation of predictive performance and the effects of feature interactions on reducing sugar yield. All models were implemented using the Scikit-learn library in Python [25]. A brief overview of each model, along with its configuration, is provided below.

• **Linear regression (LR)** [26]: The relationship between the dependent variable and the independent variables is determined by fitting a linear equation to the observed data. Advantages include simplicity and interpretability, but it cannot capture nonlinear patterns.
• **Polynomial regression (PR)** [27]: Extends LR by incorporating higher-order terms of the predictors, enabling the modeling of nonlinear relationships. In this study, second-order polynomial features were generated from the original input variables and used to train the model. While PR can fit moderate nonlinear patterns, it may overfit with higher-degree polynomials.
• **Decision tree regressor (DT)** [28]: A hierarchical, tree-like structure to partition the data based on decision rules, capturing complex, nonlinear interactions. DTs are easy to visualize but prone to overfitting. The DT model was trained with hyperparameters, with random_state= 42 to ensure reproducibility.
• **Random forest regressor (RF)** [29]: An ensemble learning method was implemented, in which multiple decision trees were constructed during training and their predictions averaged to enhance robustness and reduce overfitting. The RF model was trained using 100 estimators, each tree built on a random subset of the data. Averaging the predictions helped improve accuracy and generalization. Increasing the number of estimators generally enhances performance at the cost of higher computational time.
• **Gradient boosting regressor (GB)** [30]: An additive model was built sequentially, where each subsequent tree was trained to correct the errors of its predecessor. In this study, 100 trees were added in sequence to improve prediction accuracy. GB can capture complex nonlinearities but requires careful tuning and is more computationally intensive.
• **Artificial neural network (ANN)** [31]: A network architecture inspired by biological neural systems was employed, consisting of interconnected nodes (neurons) organized in layers to learn patterns within the data. The ANN was implemented with two fully connected hidden layers, each comprising 50 neurons and using the Rectified Linear Unit (ReLU) activation function. A maximum of 500 iterations was set for the optimization algorithm to converge during training. ANN can learn highly complex relationships but require more data and are less interpretable. Note that both PR and ANN were trained on input features normalized by their standard deviations, while the remaining models were trained on the original (unscaled) input data. To improve reproducibility and clarity, all models were implemented using Python 3.10 and the Scikit-learn library (v1.2.2) [25]. The dataset was split into training and testing sets based on hydrolysis time. The training set included data from selected hydrolysis durations at 4, 24, and 48 h and was further split into 80:20 for training and validation. The testing set comprised the remaining time points. Additionally, 5-fold cross-validation was applied to the training set to ensure model robustness. The coefficient of determination (R²) was used as the primary metric for evaluating each fold, consistent with the default scoring method in Scikit-learn for regression models. In addition to R², root mean squared error (RMSE) and mean absolute error (MAE) were calculated on the testing set to provide complementary measures of prediction accuracy.

The models’ results as well as their performance in predicting sugar concentration were evaluated using three metrics: RMSE, R^2^, and MAE. RMSE quantifies the average magnitude of prediction errors and is defined as:

(1) $$\mathrm{RMSE}=\sqrt{\frac{1}{n}\sum_{i=1}^{n}{\left(y_{i}-{\hat{y}}_{i}\right)}^{2}}$$

where $y_{i}$is the observed value, ${\hat{y}}_{i}$is the predicted value, and n is the number of observations [32]. A lower RMSE value indicates better performance of the model in predicting sugar concentration.

**The coefficient of determination (R²)** measures the proportion of variance in the dependent variable that can be explained by the independent variables as:

(2) $$R^{2}=1-\frac{\sum_{i=1}^{n}{\left(y_{i}-{\hat{y}}_{i}\right)}^{2}}{\sum_{i=1}^{n}{\left(y_{i}-{\overset{®}{y}}_{i}\right)}^{2}}$$

where $\overset{®}{y}$ is the mean of the observed values [33]. This metric indicates how well the model's predictions approximate the actual data, with a value closer to 1 representing better predictive performance.

**Mean absolute error (MAE)** calculates the average absolute differences between predicted and observed values, offering a straightforward measure of prediction accuracy [34]:

(3) $$\mathrm{MAE}=\frac{1}{n}\sum_{i=1}^{n}\left|y_{i}-{\hat{y}}_{i}\right|$$

This metric shows how close the model's predictions are to the actual values, with lower MAE values indicating better performance. Additionally, other approaches were employed to investigate the relationships between variables, including the Pearson correlation coefficient.

**Pearson correlation coefficient (PCC, r)** quantifies linear relations among continuous variables as a normalized score. The PCC (r) is between - 1 and + 1, with - 1 indicating a perfect inverse linear dependence, 0 for statistical linear independence, and + 1 indicating a good positive linear relationship [35]. The PCC (r), calculated between variables X and Y, was derived using the following:

(4) $$r=\frac{\sum_{i=1}^{N}\left(X_{i}-\overset{®}{X})(Y_{i}-\overset{®}{Y}\right)}{\sqrt{\sum_{i=1}^{N}{\left(X_{i}-\overset{®}{X}\right)}^{2}∙\sum_{i=1}^{N}{\left(Y_{i}-\overset{®}{Y}\right)}^{2}}}$$

where $X_{i}$ and $Y_{i}$ denote observed values, with $\overset{®}{X}$ and $\overset{®}{Y}$ being the averages of X and Y, respectively. The correlation coefficient r and the associated heatmap were computed and generated using Python 3.10 with Seabor (v0.13.2) and matplotlib (v3.10.0)

In addition, the Friedman test was employed to ensure that the differences among the models were statistically evaluated.

**The friedman test** is a non-parametric alternative to one-way repeated-measures ANOVA, used to detect differences among three or more paired models across multiple datasets [36]. It ranks algorithms by performance for each dataset, assessing whether observed differences are statistically significant. If significant, post-hoc tests (e.g., Nemenyi or pairwise Wilcoxon tests with multiple comparison corrections) identify which model pairs differ [37].

## 3 Results and discussion

### 3.1 Effect of acid pretreatment on sugar release from lignocellulosic biomass

Acid pretreatment is an effective chemical method for disrupting lignocellulosic biomass. It primarily solubilizes hemicellulose and partially removes lignin, thereby enhancing cellulose accessibility for subsequent enzymatic hydrolysis [38]. In most studies, pretreatment of lignocellulosic biomass is typically performed at high temperatures combined with relatively lower acid concentrations. For example, treatment with 1 % w/w H₂SO₄ at 160–180°C for 1–5 min achieved approximately 83 % sugar yield (xylose and glucose) during enzymatic hydrolysis [11]. Specific studies on different biomass types showed that pretreatment with 0.5 % H₂SO₄ at 121°C for 60 min followed by enzymatic hydrolysis resulted in sugar yields of 694 ± 2.6 mg/g for corn cob, 520 ± 1.6 mg/g for sugarcane bagasse, and 466 ± 4.2 mg/g for rice straw [39]. These findings highlight that both temperature and acid concentration are key parameters influencing sugar release, and optimal conditions can vary depending on the type of biomass and process configuration.

In this study, sulfuric acid was selected as the pretreatment agent for rice straw and sugarcane leaves, based on its widespread use in previous research [9], [10], [11]. While the exact concentrations in these studies differed, we applied concentrations of 0, 3, 6, and 9 %w/w to explore the effect of acid strength on hydrolysis efficiency in the current experimental setup.

**Fig. 1:**
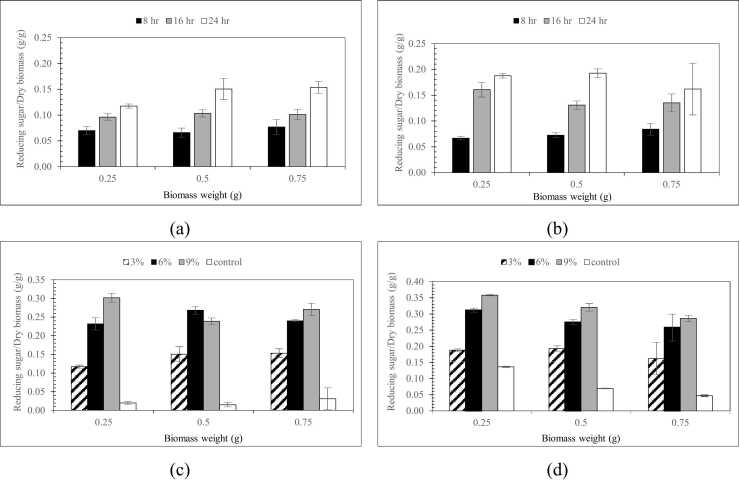
Reducing sugar yield from (a) rice straw and (b) sugarcane leaves pretreated with 3 % (v/v) sulfuric acid at different times, and from (c) rice straw and (d) sugarcane leaves after 24 h pretreatment with varying sulfuric acid concentrations.

As shown in Fig. 1, the highest acid concentration used in this experiment 9 % (w/w) at 60 °C resulted in the maximum release of reducing sugars, yielding 0.474 g/g and 0.562 g/g reducing sugar per dry biomass from rice straw and sugarcane leaves, respectively, after 24 h.

And extending the pretreatment time from 8 to 24 h enhanced sugar release, particularly at the lower biomass loading (0.25 g), whereas at 0.75 g the improvement was limited, suggesting diffusion constraints and possible sugar loss. Acid concentration exerted a stronger influence than time, with yields increasing markedly from 3 % to 9 % H₂SO₄, especially at low loading.

As shown in Fig. 1C, for 6 % H₂SO₄ at higher loadings (0.5–0.75 g) and 9 % H₂SO₄ at 0.25–0.5 g, sugar release decreased, likely due to excessive biomass loading, mass transfer limitations, and partial acid loss that restricted hydrolysis efficiency. Conversely, at 6 % H₂SO₄ (0.25–0.5 g) and 9 % H₂SO₄ (0.5–0.75 g), sugar yields increased, probably due to improved acid–substrate interaction and higher solution density, which promoted dissolution efficiency.

These nonlinear responses, frequently reported in lignocellulosic pretreatment studies [40], highlight that both underloading and overloading can reduce sugar release. Overall, acid strength and biomass load are the dominant factors, whereas extending pretreatment beyond 24 h offers only limited additional benefit.

After acid pretreatment, the cellulose becomes more accessible to cellulase enzymes, facilitating hydrolysis [41]. By reducing the structural recalcitrance of lignocellulosic material, acid pretreatment enhances biomass digestibility, resulting in higher sugar yields during the subsequent enzymatic hydrolysis step [42]. However, this process can also degrade hemicellulosic sugars, leading to the formation of toxic by-products such as acetic acid, furfural, and 5-hydroxymethylfurfural (HMF), which may inhibit microbial growth when the hydrolysate is used as a fermentation substrate [43].

### 3.2 Effect of enzymatic hydrolysis parameters

Biomass materials with high cellulose content, such as rice straw (39.58 % cellulose), yielded higher average sugar levels compared to those with lower cellulose and higher lignin content, such as sugarcane leaves (29.16 % cellulose), under identical enzymatic and hydrolysis conditions. These findings are consistent with the association between increased cellulose content and improved sugar yield from biomass hydrolysis [44]. In terms of enzyme dosage, cellulase played a critical role in sugar production. The application of cellulase alone at a dosage of 0.1 mL for rice straw yielded 0.69 g/g dry biomass at 24 h, temporarily declined to 0.48 g/g at 32 h, and increased again to 0.73 g/g at 48 h. This fluctuation may be due to transient product inhibition and subsequent reactivation as hydrolysis proceeds [45]. These results are consistent with reports indicating that an optimal amount of cellulase alone is generally more effective than enzyme mixtures in hydrolyzing rice straw [46].

While the use of mixed enzymes, a combination of 0.05 mL each of cellulase and xylanase, occasionally produced sugar yields comparable to or higher than those obtained with cellulase alone, it was often accompanied by increased variability. This variability was evident from the larger error bars (standard deviation), indicating less consistent performance. Extending the hydrolysis time from 4 to 24 or 48 h generally enhanced sugar yield. However, some 48-hour trials demonstrated markedly wider error bars, suggesting instability of the reaction environment during prolonged incubation. These fluctuations indicated instability in the reaction environment over prolonged periods, potentially resulting from enzyme degradation or the accumulation of inhibitory compounds. Consequently, the magnitude of the error bars served as a practical indicator of process stability: small deviations reflected reproducible outcomes, whereas large deviations, even alongside high mean sugar yields, suggested potential uncertainty in process performance. This distinction was critical for optimizing conditions in industrial-scale enzymatic hydrolysis.

### 3.3 Surface morphology analysis results by Scanning Electron Microscope (SEM analysis)

**Fig. 2:**
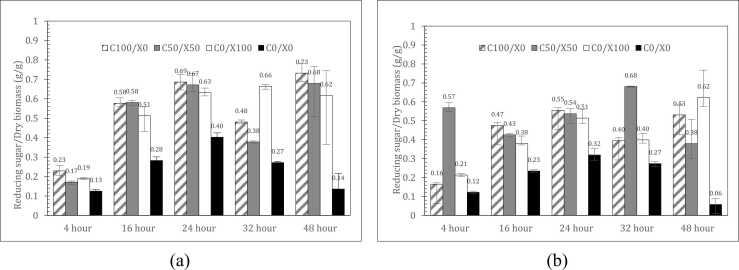
Enzymatic hydrolysis performance showing reducing sugar production (g/g) from (a) rice straw and (b) sugarcane leaves at varying ratios of C (cellulase) and X (xylanase).

**Fig. 3:**
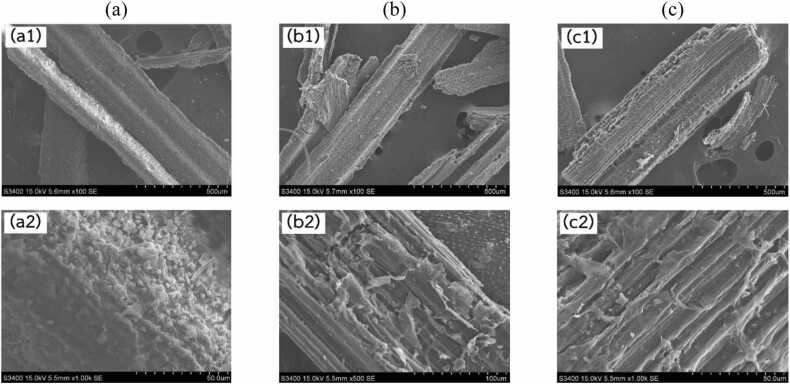
Scanning electron microscope (SEM) images showing the surface morphology of rice straw: (a) untreated raw material (a1-unmagnified, a2-magnified), (b) after sulfuric acid pretreatment (b1-unmagnified, b2-magnified), and (c) after sequential acid pretreatment and enzymatic hydrolysis (c1-unmagnified, c2-magnified).

SEM images from Fig. 2, Fig. 3 were employed to examine the surface morphology and complex fiber structures of solid biomass materials. Detailed SEM analysis revealed notable morphological changes in rice straw samples subjected to different pretreatment conditions. In image (a), the raw rice straw exhibited well-organized fibrous structures and intact plant cell walls, suggesting that the fibers were still coated with lignin. In image (b), after sulfuric acid pretreatment, the structure appeared distorted and significantly disordered, with disrupted cell walls. This disruption is likely due to the partial removal of lignin, which may have facilitated increased enzyme accessibility to the cellulose matrix, thereby enhancing the rate of enzymatic hydrolysis. In image (c), the rice straw subjected to combined acid and enzymatic pretreatment displayed a porous structure, indicating that enzymatic hydrolysis had utilized the cellulose, creating visible pores. The observed morphological alterations align with the influence of acid pretreatment in disrupting lignin-carbohydrate linkages and lower cellulose crystallinity, thereby enhancing enzymatic hydrolysis [47]*.*

**Fig. 4:**
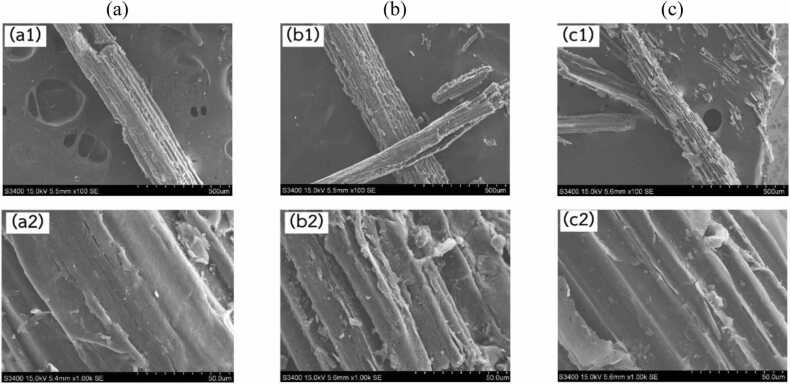
Scanning electron microscope (SEM) images showing the surface morphology of sugarcane leaves: (a) untreated raw material (a1-unmagnified, a2-magnified), (b) after sulfuric acid pretreatment (b1-unmagnified, b2-magnified), and (c) after sequential acid pretreatment and enzymatic hydrolysis (c1-unmagnified, c2-magnified).

In addition, a sequential pretreatment using acid followed by enzymatic hydrolysis resulted in the highest sugar release. For example, rice straw subjected to this sequential process achieved a glucose-to-ethanol conversion efficiency of 97.68 %, highlighting the efficacy of the combined pretreatment approach in enhancing fermentable sugar recovery for bioethanol production [48]*.* Fig. 4 shows the morphological changes observed in sugarcane leaf samples. In (a), the fiber structure is grouped and well-organized, with an intact plant cell wall surface, indicating the presence of lignin coating the fibers. Lignin, a complex phenolic polymer, imparts structural strength and hydrophobic properties to the cell walls of plants. It acts as a barrier that restricts enzymatic access to cellulose and hemicellulose. This description helps frame our observation regarding the relatively high lignin concentration found in sugarcane leaves. The intact lignin–carbohydrate matrix likely contributes to the decreased enzymatic digestibility seen in untreated or minimally processed sugarcane leaves, reinforcing our hypothesis that elevated lignin levels obstruct sugar release. Consequently, the success of chemical or microbial pretreatments is closely tied to the composition and bonding of lignin within the biomass structure [49], [50], [51]. Studies confirm that sugarcane leaves have a high lignin content (14–20 %), which contributes to their recalcitrance [52].

In (b), the structure appears disrupted, with open and disordered cell walls after sulfuric acid pretreatment, likely due to the partial removal of lignin. Acid treatment reduces xylan content and modifies lignin composition, as observed in sugarcane bagasse studies, where dilute sulfuric acid removed hemicellulose and acid-soluble lignin [52]. In (c), the sugarcane leaf sample, after enzymatic hydrolysis, exhibited structural degradation characterized by pore formation, indicating that enzymatic activity primarily disrupts the biomass structure and facilitates the hydrolytic breakdown of individual cellulose fibers [53].

### 3.4 Input features correlation analysis

**Fig. 5:**
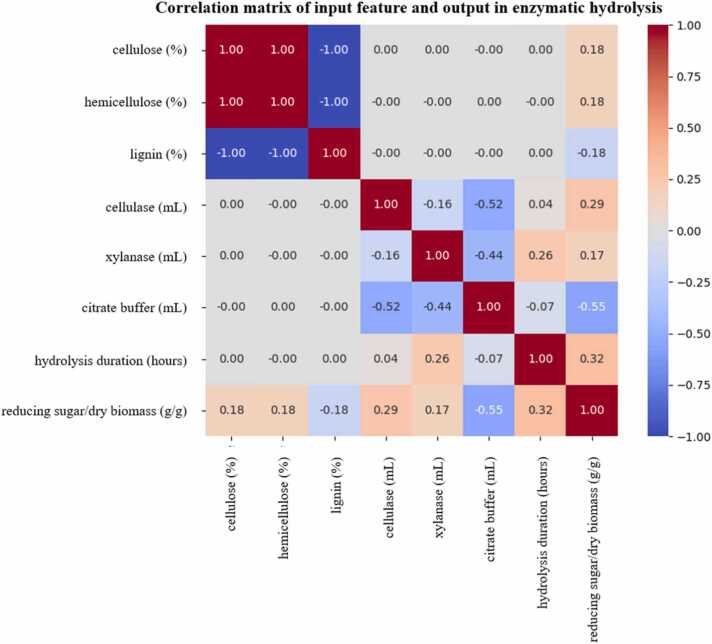
Correlation matrix of input variables used in enzymatic hydrolysis modeling.

To study the relationships among input features in enzymatic hydrolysis, a correlation analysis was performed in Fig. 5. Cellulose, hemicellulose, and lignin had linear correlations (r = ±1.00), reflecting proportional interdependence of the biomass composition. Specifically, cellulose and hemicellulose were positively correlated with each other (r = 1.00), while both showed negative correlations with lignin (r = –1.00). This structural limitation has the potential for multicollinearity, which can undermine model interpretability and stability but does not imply a fixed stoichiometric ratio and can vary widely depending on species, growth conditions, and environmental factors [54]. Therefore, the correlations observed in this dataset likely arise from structural constraints in the compositional data rather than from universal stoichiometric relationships in biomass. The impact of this multicollinearity varies across the models. For Linear Regression, perfect multicollinearity can cause the model to be unstable, and the coefficient estimates to be unreliable or highly inflated [55]. However, this effect is largely mitigated in our ensemble tree-based models (RF and GB), which are inherently robust to multicollinearity. This is because these models select features based on their individual information gain and don't rely on the inversion of a feature matrix like linear models do. The robustness of tree-based methods to multicollinearity is an advantage [56].

For the output variable, reducing sugar per dry biomass, a slight positive correlation was observed with cellulose (r = 0.18), cellulase (r = 0.29), xylanase (r = 0.17), and hydrolysis duration (r = 0.32). These trends indicate that higher cellulose content, enzyme dosages, and longer reaction times may be associated with somewhat increased reducing sugars from lignocellulosic biomass [57]. In particular, the higher correlations observed for cellulase and hydrolysis time indicate their important roles in facilitating the enzymatic breakdown of cellulose and hemicellulose to sugars [58].

In contrast, reducing sugar per dry biomass was negatively correlated with lignin (r = –0.18) and buffer volume (r = –0.55). This suggests that high lignin content may hinder enzymatic hydrolysis by acting as a physical barrier, preventing effective enzyme access to cellulose and hemicellulose [59]. Similarly, a large buffer volume may reduce the relative concentration of enzymes or alter optimal reaction conditions (e.g., pH), thereby negatively affecting sugar release efficiency.

These correlation patterns are consistent with the known biochemical mechanisms of enzymatic hydrolysis and help to understand the relationships between variables, which can aid in data exploration and model fitting, but do not directly indicate optimization strategies for enhancing sugar yield from lignocellulosic feedstocks.

### 3.5 Sugar concentration prediction results

**Model validation of predicted and experimental sugar concentrations.** To demonstrate the generative capability of the machine learning approach, the models were used to predict reducing sugar concentrations under unseen conditions. The models: LR, PR, DT, RF, GB, and ANN, were trained using experimental data collected at 4, 24, and 48 h. They were then tested on unseen data at 16 and 32 h with varying cellulase/xylanase ratios (100/0, 50/50, 0/100, and 0/0) for both rice straw and sugarcane leaves. The predicted values and corresponding experimental values are presented in Table 2.

**Table 2:** Comparison of predicted and experimental reducing sugar for the enzymatic hydrolysis stage.

**Biomass**	**Cellulase/** **xylanase ratio**	**Time (h)**	**Reducing sugar/dry biomass (g/g)**						
			**Predicted value**						**Experimental value**
			**LR**	**PR**	**DT**	**RF**	**GB**	**ANN**	
**Rice straw**	100/0	16	0.5138	0.5300	0.6862	0.6856	0.6750	0.4944	0.5771
	50/50		0.4783	0.5374	0.6422	0.6407	0.6484	0.3807	0.5813
	0/100		0.4429	0.4886	0.6351	0.6334	0.6397	0.4773	0.5130
	0/0		0.1865	0.2859	0.4091	0.3821	0.3890	0.2204	0.2827
**Sugarcane** **leaves**	100/0	16	0.4331	0.4823	0.5480	0.5507	0.5510	0.3909	0.4750
	50/50		0.3976	0.4948	0.5370	0.5345	0.5463	0.5239	0.4257
	0/100		0.3622	0.4511	0.5143	0.5105	0.5071	0.3834	0.3799
	0/0		0.1058	0.2711	0.3022	0.2906	0.3105	0.2759	0.2348
**Rice straw**	100/0	32	0.5760	0.7429	0.6862	0.6856	0.6750	0.6849	0.4806
	50/50		0.5405	0.7226	0.6422	0.6407	0.6484	0.6175	0.3778
	0/100		0.5051	0.6460	0.6315	0.6334	0.6397	0.5456	0.6644
	0/0		0.2487	0.3544	0.4092	0.3822	0.3890	0.2273	0.2721
**Sugarcane** **leaves**	100/0	32	0.4953	0.5633	0.5480	0.5507	0.5510	0.5401	0.3954
	50/50		0.4598	0.5481	0.5370	0.5345	0.5463	0.4610	0.6806
	0/100		0.4244	0.4766	0.4143	0.5105	0.5071	0.5183	0.3989
	0/0		0.1680	0.2078	0.3022	0.2874	0.3105	0.1767	0.2736

**Fig. 6:**
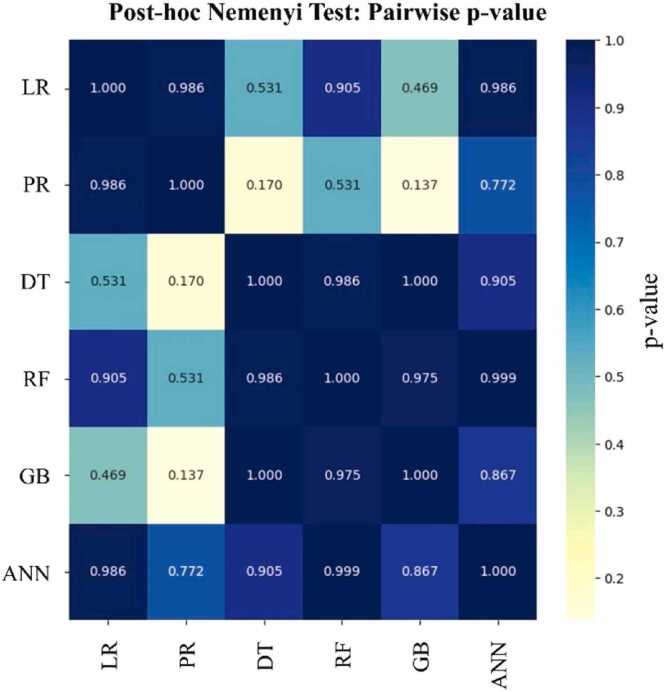
Post-hoc Nemenyi test showing pairwise p-values among prediction models, illustrating which model pairs exhibit statistically significant differences in performance.

The results showed that tree-based models (DT, RF, and GB) demonstrated strong overall performance and were able to capture nonlinear relationships between the input variables. Similarly, the ANN model showed prediction trends consistent with the experimental outcomes in several scenarios. Its performance was sometimes unstable, due to the limited dataset size and sensitivity to hyperparameter settings, which may lead to overfitting if not properly optimized. In contrast, the simpler models (LR and PR), despite their limited complexity, often yielded reducing sugar values closer to the experimental data with lower prediction errors in certain conditions, as further illustrated in Fig. 6. This indicates that while regression-based approaches may not fully capture the intricate interactions in biomass hydrolysis, they remain valuable for interpretability and in cases where tree-based models overfit.

In summary, selecting an appropriate predictive model for reducing sugar yield from enzymatic hydrolysis of lignocellulosic biomass should consider both data complexity and the trade-off between predictive accuracy and interpretability. While LR and PR often underestimated reducing sugar values, in many cases, they provided more accurate predictions than complex methods such as ANN and tree-based models. For example, for rice straw C0/X100, the experimental value was 0.5130, whereas LR and PR yielded 0.4429 and 0.4886, respectively, which were closer than the tree-based predictions (∼0.63).

Based on models trained with data at 4, 24, and 48 h, predictions were evaluated at unseen time points (16 and 32 h). While Table 2 shows that DT performed the best in the training set, LR extrapolated better to new conditions. This outcome means that simpler models, although they are limited, generalize better to unseen data. At the same time, more sophisticated methodologies such as tree-based and ANN may be dataset-size and hyperparameter-setting sensitive. Overall, these results emphasize the importance of balancing model complexity with the ruggedness of prediction in selecting methods for reducing sugar prediction.

#### 3.5.1 Model performance evaluation

To demonstrate the models’ performance in predicting sugar concentrations, Table 3 presents the R², RMSE, and MAE values for all models. The results are based on training with data collected at 4, 24, and 48 h of the enzymatic hydrolysis process and testing with data from 16 and 32 h. The combination of R², RMSE, and MAE provides a comprehensive evaluation of model performance. R² reports the proportion of variance explained by the model, RMSE penalizes larger errors by squaring differences, and MAE provides an interpretable mean prediction error. Together, these statistics confirm that the DT model generates accurate and reliable predictions for sugar concentration.

**Table 3:** Comparison of RMSE, MAE, and R² for regression models at multiple enzymatic hydrolysis time points (models are trained with data from 4, 24, and 48 h of enzymatic hydrolysis processes).

**Models**	**RMSE**		**MAE**		**R**^**2**^	
	**Train**	**Test**	**Train**	**Test**	**Train**	**Test**
Linear Regression	0.1642	0.1773	0.1419	0.1560	0.4249	0.4564
Polynomial Regression	0.1051	0.1537	0.0776	0.1124	0.7644	0.5917
Decision Tree	0.0715	0.1042	0.0433	0.0705	0.8910	0.8121
Random Forest	0.0741	0.1150	0.0483	0.0820	0.8830	0.7712
Gradient Boosting	0.0759	0.1134	0.0518	0.0756	0.8771	0.7777
Artificial Neural Network	0.1009	0.1364	0.0821	0.1155	0.7828	0.6784

Among all evaluated models, the DT achieved the highest prediction accuracy, with an R^2^ of 0.8121, accompanied by the lowest RMSE and MAE of 0.1042 and 0.0705, respectively. In comparison, other models such as LR, PR, RF, GB, and ANN exhibited lower R² values and higher error metrics.

The minimal difference between training and testing performance indicates the robustness of the DT model and limited overfitting, demonstrating its ability to capture complex and nonlinear relationships in the data. These results show that while DT is effective for modeling measured data, simpler models can generalize better to new conditions, emphasizing the need to balance model complexity with predictive robustness in sugar yield prediction.

#### 3.5.2 Model selection for sugar concentration prediction

The Friedman test did not show a statistically significant difference among the models (p ≥ 0.05). The post-hoc Nemenyi Test was conducted to prove the effectiveness of all six models on the unseen test (hydrolysis time at 16 and 31 h). Fig. 6 displays the pairwise p-values, where each value represents the p-value of the comparison between the model in the row and the model in the column. At a significance level (α) of 0.05, it was found that all p-values were greater than 0.05, except for the diagonal values of 1.0, which indicate a model being compared with itself.

For example, the p-value for the comparison between PR and GB was 0.137, which is greater than 0.05. The lowest p-value was 0.137, which is still higher than the typical threshold. Therefore, there is no statistically significant difference in performance between any pair of models at the conventional 0.05 significance level. This means that any of the models can be used interchangeably for prediction.

In addition, PR and LR provided acceptable prediction accuracies, reflecting their limitations in modeling complex nonlinear interactions, which may have resulted in higher error metrics and reduced performance compared to tree-based models. Although ANN have the potential to learn complex relationships, the model proved effective for predicting sugar yields [15]. The ANN results showed substantially lower R² values and higher errors relative to tree-based methods. This may be attributed to the higher data requirements and the complexity of parameter tuning, which were not fully optimized in the present study.

Although the DT model demonstrated high performance (R² = 0.8480, RMSE = 0.0792, MAE = 0.0566), it cannot provide explicit mathematical equations. Nevertheless, LR and PR offer an interpretable alternative, as presented in (4), (5), respectively. Furthermore, in Fig. 6, these simpler models yield predictions reasonably close to the experimental values.

(5) $$\hat{y}=399.10-9.83x_{1}+4.54x_{2}-7.36x_{3}-0.48x_{4}-0.54x_{5}-3.08x_{6}+0.01x_{7}$$

(6) $$\hat{y}=-1.04\times 10^{4}\left[\begin{array}{c} \left(0.13x_{1}-0.07x_{2}+0.12x_{3}-0.61x_{4}-1.11x_{5}\;+1.59x_{6}+0.04x_{7}\right)+\\ \left(6.12x_{1}^{2}-3.63x_{2}^{2}+4.65x_{3}^{2}-3.84x_{4}^{2}-3.92x_{5}^{2}+31.18x_{6}^{2}-3.77\times 10^{-4}x_{7}^{2}\right)+\\ \left(-0.27x_{2}+7.88x_{3}+19.42x_{4}+33.05x_{5}-53.90x_{6}-0.45x_{7}\right)x_{1}+\\ \left(1.51x_{3}-25.02x_{4}-44.18x_{5}+66.39x_{6}+1.2x_{7}\right)x_{2}+\\ \left(4.69x_{4}+6.97x_{5}-14.58x_{6}+0.28x_{7}\right)x_{3}+\\ \left(3.77x_{5}-5.98x_{6}-1.89x_{7}\right)x_{4}+\left(-10.78x_{6}-1.91x_{7}\right)x_{5}+(-1.96x_{6})x_{7} \end{array}\right]$$

where x_1_, x_2,_ and x_3_ represent the cellulose, hemicellulose, and lignin content (%) respectively; x_4_, x_5,_ and x_6_ denote the volumes (mL) of cellulase, xylanase, and citrate buffer used; and x_7_ corresponds to the hydrolysis time (hours); and $\hat{y}$ is the predicted value of reducing sugar (g/g dry biomass). In summary, tree-based models, particularly the DT, demonstrated superior suitability for predicting reducing sugar yield in enzymatic hydrolysis processes. Their ability to handle nonlinearities and complex data structures with high accuracy and robustness offers a significant advantage for experimental planning and process optimization.

## 4 Conclusions

Biomass loading was found to significantly affect sugar yield. Longer pretreatment times, particularly under sequential acid pretreatment followed by enzymatic hydrolysis, further enhanced sugar release. Although ANN was initially employed to identify optimal hydrolysis conditions, the DT model ultimately demonstrated superior predictive accuracy, achieving R² values of 0.8910 (training) and 0.8121 (testing), with low RMSE and MAE. The DT model was able to capture complex nonlinear relationships within the dataset, although the predictive performance is subject to the limitations of the relatively small dataset. However, LR can make predictions for unseen time points. This shows the distinction between apparent statistical metrics and practical predictive accuracy. Simpler models like LR capture the underlying linear trends and generalize better, whereas complex models like DT may overfit the limited training data, reflecting the classic bias-variance tradeoff in machine learning. A notable limitation of tree-based models is their inability to provide explicit mathematical equations. Therefore, LR and PR, as shown in (4), (5) respectively, remain valuable for interpretability, and their predictions during validation were reasonably close to the experimental values. While ANN showed comparatively lower accuracy in this study, its performance could likely be improved through careful hyperparameter optimization, leveraging its inherent ability to model complex relationships. We recommend exploring such optimization in future work to enhance predictive performance. These advantages align with supporting the development of more sustainable biomass conversion processes.

## CRediT authorship contribution statement

**Kittisak Ngowsakul:** Methodology, Investigation, Formal analysis. **Namboonlue Supitchayanee:** Writing – review & editing, Writing – original draft, Visualization, Validation, Software, Methodology, Investigation, Formal analysis, Data curation, Conceptualization. **Tunyaboon Laemthong:** Writing – review & editing, Writing – original draft, Validation, Resources, Project administration, Methodology, Investigation, Funding acquisition, Formal analysis, Data curation, Conceptualization. **Pakorn Uttayopas:** Writing – review & editing, Methodology, Investigation, Formal analysis. **Nakarin Subjalearndee:** Writing – review & editing, Investigation. **Chatchol Kongsinkaew:** Writing – review & editing, Investigation. **Kittiya Nakarat:** Methodology, Investigation. **Theppanya Charoenrat:** Writing – review & editing, Resources, Methodology, Validation.

## Declaration of Competing Interest

The Authors declare that there are no competing interests associated with the manuscript.
